# Investigating developmental cardiovascular biomechanics and the origins of congenital heart defects

**DOI:** 10.3389/fphys.2014.00408

**Published:** 2014-10-21

**Authors:** William J. Kowalski, Kerem Pekkan, Joseph P. Tinney, Bradley B. Keller

**Affiliations:** ^1^Cardiovascular Innovation Institute, University of LouisvilleLouisville, KY, USA; ^2^Department of Pediatrics, University of LouisvilleLouisville, KY, USA; ^3^Department of Biomedical Engineering, Carnegie Mellon UniversityPittsburgh, PA, USA

**Keywords:** biomechanics, cardiovascular development, computational modeling, congenital heart disease, embryo, hemodynamics

## Abstract

Innovative research on the interactions between biomechanical load and cardiovascular (CV) morphogenesis by multiple investigators over the past 3 decades, including the application of bioengineering approaches, has shown that the embryonic heart adapts both structure and function in order to maintain cardiac output to the rapidly growing embryo. Acute adaptive hemodynamic mechanisms in the embryo include the redistribution of blood flow within the heart, dynamic adjustments in heart rate and developed pressure, and beat to beat variations in blood flow and vascular resistance. These biomechanically relevant events occur coincident with adaptive changes in gene expression and trigger adaptive mechanisms that include alterations in myocardial cell growth and death, regional and global changes in myocardial architecture, and alterations in central vascular morphogenesis and remodeling. These adaptive mechanisms allow the embryo to survive these biomechanical stresses (environmental, maternal) and to compensate for developmental errors (genetic). Recent work from numerous laboratories shows that a subset of these adaptive mechanisms is present in every developing multicellular organism with a “heart” equivalent structure. This chapter will provide the reader with an overview of some of the approaches used to quantify embryonic CV functional maturation and performance, provide several illustrations of experimental interventions that explore the role of biomechanics in the regulation of CV morphogenesis including the role of computational modeling, and identify several critical areas for future investigation as available experimental models and methods expand.

## Introduction

The heart and vasculature is the first organ system to form and the only one which is required to function successfully throughout embryonic and fetal life for survival (Burggren and Keller, 1998). It is also the organ system where congenital defects are associated with the highest incidence and morbidity and mortality and adult onset diseases are the greatest cause of medical costs and mortality (Go et al., 2013). In the US, congenital heart disease (CHD) occurs in at least 8 of every 1000 live births and accounts for more than 24% of birth defect related infant deaths (Go et al., 2013). Due to this prevalence, cardiovascular (CV) morphogenesis and the origins of CHD have been widely researched for centuries. This work has uncovered a variety of molecular and cellular mechanisms, morphogenetic and structural processes, and biomechanical cues responsible for the formation of the CV. In turn, this understanding has enabled dramatic advances in the treatment and management of CHD, improving survival rates to 90% and resulting in over 1 million adults with successfully treated CHD in the US alone.

CV development is highly conserved among vertebrate species. As a result, a variety of animal models are employed to study CV development based on the unique technologies employed and the questions to be answered. These models include the zebrafish, frog, chick, and mouse (Nieuwkoop and Faber, 1994; Lohr and Yost, 2000; Isogai et al., 2001; Glickman and Yelon, 2002; Levine et al., 2003; Martinsen, 2005; Bruneau, 2008; Savolainen et al., 2009; Al Naieb et al., 2012). In the vertebrate embryo, bilateral endocardial tubes fuse at the ventral midline of the embryo to form the primitive linear heart tube, which begins to beat. Cardiac looping transforms the heart into a looped tube while later events include the formation of endocardial cushions, septation, ventricular trabeculation, and remodeling of the heart valves. Both avians and mammals develop a fully septated, four-chambered heart. A comparative timeline among vertebrate species is given in Table 1. Frog embryos are staged according to Nieuwkoop-Faber (NF) and chick embryos according to Hamburger-Hamilton (HH) (Hamburger and Hamilton, 1951; Nieuwkoop and Faber, 1994).

**Table 1:** **Comparative timeline of events in cardiovascular development expressed as days post fertilization**.

	**Zebrafish**	**Frog (NF stages)**	**Mouse**	**Chick (HH stages)**	**Human (Carnegie)**
Gestation/Incubation period			20 days	21 days	9 months
Linear heart tube	1	1.67 (32)	8	1.29 (9)	28 (10)
First heartbeat	1	1.83 (33)	8	1.29 (9)	28 (10)
Onset of blood flow	1	1.83 (33)	8.5	1.46 (10)	29 (11)
Cardiac looping	1–2	1.67–2.75 (32–40)	8–10.5	1.46–4 (10–24)	28–32 (10–13)
Onset of ventricular trabeculation	2	3.17 (41)	9.5	2.23 (16)	29 (11)
Atrial septation		3.83–4.42 (44-46)	10–14.5	2.23–10 (16–36)	30–46 (12–19)
Ventricular septation			9–14	2.42–8(17–34)	30–46 (12–19)
Atrioventricular cushions form	2	2.33 (39)	10	2.23 (16)	32 (13)
Outflow tract cushions form		2.33 (39)	10	3.23 (19)	32 (13)

*The numbers in parentheses represents standardized stages, if applicable. Sissman, 1970; O'rahilly, 1971; O'rahilly et al., 1987; Nieuwkoop and Faber, 1994; Lohr and Yost, 2000; Isogai et al., 2001; Glickman and Yelon, 2002; Levine et al., 2003; Martinsen, 2005; Bruneau, 2008; Savolainen et al., 2009; O'rahilly and Muller, 2010; Al Naieb et al., 2012*.

As blood flows through the vascular system, it exerts normal force due to pressure, circumferential stress due to vessel deformation under pressure, and a tangential wall shear stress (WSS) due to pulsatile blood flow. Over a century ago, based on observations of chick embryos, Thoma proposed that these forces influence vascular morphology (Thoma, 1893). Since that time, vascular adaptation in response to blood flow, or the “flow dependency principle” has been established in the mature CV system (Kamiya and Togawa, 1980). Increased transmural stress due to hypertension elicits a remodeling response including wall thickening, increased smooth muscle proliferation, and increased collagen production and turnover (Nissen et al., 1978; Olivetti et al., 1980; Fung and Liu, 1991; Tedgui et al., 1992; Intengan et al., 1999a,b). Experiments in a variety of species to alter blood flow within vessels demonstrate that increased flow results in increased diameter and decreased flow results in decreased diameter or vessel regression (Kamiya and Togawa, 1980; Guyton and Hartley, 1985; Langille and O'donnell, 1986; Girerd et al., 1996; Bayer et al., 1999; Gruionu et al., 2005). Mural growth and remodeling act to return wall stress to normal values (Fung and Liu, 1991) while luminal growth returns WSS to levels experienced prior to the flow alteration (Kamiya and Togawa, 1980; Langille and O'donnell, 1986). It is thought that hemodynamic forces deform the vascular wall and in particular the endothelial cells, which respond by transducing biochemical signals leading to morphological and structural changes (Chien, 2007). Likewise, early embryonic endothelial cell function depends on pulsatile blood flow (Burggren, 2013).

The relationship between CV development, morphogenesis, and biomechanical events is an area of active investigation (Clark and Hu, 1982; Le Noble et al., 2004; Lucitti et al., 2007). Mechanical, pharmacological, and genetic models that disrupt or eliminate blood flow have been used to demonstrate the requirement of hemodynamic forces for CV development (Hogers et al., 1999; Sedmera et al., 1999; Bartman et al., 2004; Vermot et al., 2009; Karunamuni et al., 2014). However, a paucity of quantitative morphometric and hemodynamic data limits our understanding of the phenotypic response. Molecular studies have identified some genes and signaling pathways involved in the hemodynamic response (Groenendijk et al., 2005; Yashiro et al., 2007; Egorova et al., 2011), but requires more research to delineate these relationships. We believe that a multidisciplinary approach is essential to incorporate all of the elements required to generate valid representations of the complex biology reflected in CV morphogenesis within adaptive and predictive computational models relevant to human CHD and to identify novel molecular regulatory threshold involved in development and remodeling (Pekkan and Keller, 2013).

Advanced methods of measuring cardiac function, improved imaging modalities enabling synchronus, high-resolution and three-dimensional (3D) live imaging, enhanced molecular techniques, and novel experimental interventions enable complementary, quantitative studies to link phenotypic growth, morphogenesis, and remodeling events with hemodynamic forces and fundamental biologic mechanisms. This review presents an overview of approaches to study hemodynamics and CV morphogenesis in the embryo and how they have contributed to our current understanding of developmental CV biomechanics. The development of computational models to investigate higher-order flow characteristics and mechanical loading is also addressed. Finally, the outlook for future research is discussed.

## Imaging techniques

Classical studies of embryonic CV morphology were performed under microscopic observation and through thin tissue sections, creating several monographs on the subject (Patten, 1920; Hughes, 1934; Romanoff, 1960; Theiler, 1972). In recent years, serial tissue sections have been combined with *in situ* hybridization to create 3D reconstructions of the embryonic chick heart with segmented tissue structures (Van Den Berg and Moorman, 2011). Scanning electron microscopy (SEM) creates 3D surface renderings of the heart and vasculature, aiding in describing events such as cardiac looping (Manner, 2009). While tissue sections and SEM are useful for these descriptive studies, quantitative morphometry is limited due to the fixation processes. Recently, episcopic fluorescence image capture (EFIC) has been combined with cryo-embedding to obtain 3D geometries of the fetal mouse with minimal tissue distortion, allowing some quantitative analysis (Yap et al., 2014). These ex vivo approaches, wherein the embryo is fixed and removed from its developmental environment, are useful for understanding overall anatomy, but cannot provide information on dynamic biomechanical events. In the next sections, we review *in vivo* techniques that allow imaging of live embryos.

*In vivo* embryonic imaging has become widely used to understand embryonic CV morphogenesis both qualitatively and quantitatively. Various modalities are currently employed, summarized in Table 2 (Gregg and Butcher, 2012). The fluorescent stereomicroscope remains a prominent tool for experiments and observation, including assessment of CV dimensions and tracking intracardiac flow patterns (Faber et al., 1974; Keller et al., 1990, 1996; Hogers et al., 1995; Al Naieb et al., 2012; Kowalski et al., 2014). Video microscopy has been used to measure ventricular epicardial surface-strain relations when combined with microspheres as fiducial markers (Tobita et al., 2002). However, these techniques are limited to tissue surfaces. Confocal microscopy offers high resolution, but is limited by shallow depth of view and primarily used in early stage embryos (Zamir et al., 2006; Yalcin et al., 2010). The small size and transparency of zebrafish can overcome some of these limitations, and transgenic zebrafish models have been combined with live confocal microscopy to analyze cardiac and blood flow dynamics (Forouhar et al., 2006; Corti et al., 2011). Multi-photon microscopy (MPM) combined with long working distance stereomicroscopy offers greater imaging depth than traditional confocal and provides 3D time-lapse microstructural images. Multi-photon 2nd harmonic auto-fluorescence collagen and elastin *in vivo* imaging protocols are currently being used to complement standard microstructural immunohistochemistry (Robertson et al., 2012).

**Table 2:** **Comparison of *in vivo* imaging modalities**.

	**Resolution (μm)**	**Depth of Field (mm)**
Confocal	1	0.2
MPM	1	2
micro-CT	1–25	80
MRM	30	100
High frequency ultrasound	30	35
OCT	4	2

Micro-computed tomography (micro-CT) offers the greatest theoretical resolution and has been widely used to obtain 3D geometries of fixed tissues through contrast agents or polymeric casting of vascular structures (Butcher et al., 2007b; Kim et al., 2011; Wong et al., 2012). *In vivo* imaging is possible with micro-CT, but has been limited to date (Henning et al., 2011). Magnetic resonance microscopy (MRM) is another emerging high-resolution, *in vivo* modality (Bain et al., 2007; Holmes et al., 2009). Both micro-CT and MRM provide opportunity for quantitative morphometric analysis.

Ultrasound imaging of the mouse embryo was initially reported in 1995 by Turnbull et al. using an ultrasound backscatter microscope to study the embryonic brain in utero between embryo days 9.5 and 11.5 at a resolution of 50 microns (Turnbull et al., 1995). The following year, Gui et al. published the first application of ultrasound to quantify cardiac function in the normal and abnormal mouse embryonic heart (Gui et al., 1996; Zhou et al., 2003). This technology became incorporated into multiple paradigms to screen for congenital defects in mutagenesis screens for congenital heart defects (Yu et al., 2004) and to quantify changes in embryonic CV function in response to maternal environmental factors (hypoxia, Furukawa et al., 2007; lithium, Chen et al., 2008; caffeine, Momoi et al., 2008) and in response to altered genes critical to cardiac morphogenesis (Zhou et al., 2005).

In recent years, optical coherence tomography (OCT) has emerged as a preferred method for live imaging of the chick embryo (Yelbuz et al., 2002; Davis et al., 2008; Manner et al., 2008). OCT is non-invasive and the 4 μm resolution is on the scale of micro-CT and greater than MRM (Yelbuz et al., 2002; Zhang et al., 2003; Kim et al., 2011). The scan rate of OCT is near 100 2D frames per second, sufficient to capture the dynamic cardiac cycle of chick embryos. OCT is limited by light scattering in embryonic tissue and has a depth of view of 1–2 mm. The speed and non-invasive aspects of confocal microscopy and OCT have been leveraged to enable long-term, time-lapse imaging (Kamei and Weinstein, 2005; El-Ghali et al., 2010; Kulesa et al., 2010; Happel et al., 2011).

Despite these *in vivo* techniques, quantitative analysis of CV morphogenesis remains lacking. To understand the influence of biomechanical cues such as hemodynamic force, a greater knowledge-base of CV morphometry is required. In particular, comparisons between normal and experimentally perturbed CV morphogenesis cannot occur without quantitative phenotypic data. Long-term imaging techniques offer a unique approach, as they provide spatially and temporally resolved data, which can be quantitatively analyzed. Previous studies using micro-CT (Henning et al., 2011) and MRM (Bain et al., 2007; Holmes et al., 2009) have followed single embryos over a period of days to quantify organ volume growth and changes in cardiac function. Embryos were imaged at set intervals and returned to the incubator in the interim. The imaging environment varied among the different studies from room air (Bain et al., 2007) to full temperature and humidity control (Henning et al., 2011). Long-term, time-lapse imaging of live embryos, where a single embryo is kept within an imaging platform and followed continuously for a period of hours or days, has been achieved by constructing controllable chambers to maintain physiologic environmental conditions (Orhan et al., 2007; Gargesha et al., 2009; Kulesa et al., 2010; Ma et al., 2010; Happel et al., 2011; Al Naieb et al., 2012). The chick embryo has been studied using long-term, time-lapse techniques combined with both confocal microscopy to track moving cell populations over a period of more than 26 h, in 1.5 min intervals (El-Ghali et al., 2010; Kulesa et al., 2010) and OCT to measure cardiac function over a 6 h period, in 60 min intervals (Happel et al., 2011). A confocal system has been developed for long-term, time-lapse imaging of the zebrafish embryo as well, capable of a 5 day period, in 10 min intervals (Kamei and Weinstein, 2005). A challenge of these continuous techniques is maintaining the embryo in an exposed and stationary state to facilitate imaging. Shell-less or *ex ovo* culture systems are often used in long-term studies of chick embryos (Teddy et al., 2005; El-Ghali et al., 2010; Happel et al., 2011), though an *in ovo* confocal protocol has been proposed (Kulesa et al., 2010). In the case of zebrafish embryos, immobile mutants or tricaine anesthetized embryos are used (Kamei and Weinstein, 2005). The effects of these conditions on embryonic development should be addressed when designing the experiment and analyzing the data. For example, shell-less culture of chick embryos has been associated with changes in heart rate and developmental delay compared to *in ovo* embryos (Auerbach et al., 1974; Yelbuz et al., 2000).

## Cardiac function measurements

### Intracardiac flow patterns

Early investigations of the hemodynamic influence on CV development focused on intracardiac flow patterns. Flow patterns in the chick embryo were described through observation of the erythrocytes, and led to a widely perceived notion that a spiraling pattern of flow streams determined cardiac septation (Bremer, 1932; Jaffee, 1965). Dye injections and improved microscopy techniques of later researchers disproved these conclusions, but maintained that separate flow streams exist in the embryonic heart (Yoshida et al., 1983; Hogers et al., 1995). Intracardiac flow patterns depend on both the site of origin and embryonic stage (Rychter and Lemez, 1965; Yoshida et al., 1983; Hogers et al., 1995). Although these patterns do not predict the eventual location and shape of the septa, their disruption does result in a variety of cardiovascular defects including ventricular septal defects, bicuspid aortic valve, and anomalies of the great arteries (Hogers et al., 1999), suggesting that these flow streams provide some environmental cues for cardiac morphogenesis.

### Blood flow velocity

As embryonic development proceeds, cardiac physiology changes rapidly. Measurements of cardiac function are primarily performed in chick embryos, as they are amenable to instrumentation. For decades, pulsed-Doppler ultrasound has been the standard technique for velocity, stroke volume, and cardiac output measurements in the embryo (Hu and Clark, 1989; Broekhuizen et al., 1993; Phoon et al., 2000; Ursem et al., 2001). Doppler ultrasound measures the phase shift between two consecutive pulses to determine velocity of moving particles, such as red blood cells. The Doppler shift is measured in the direction of the ultrasound beam, and is therefore a 1D measurement (Zheng et al., 2006). The angle between the ultrasound beam and vessel centerline, the Doppler angle, is used to compute velocity. Measurement of the vessel diameter is combined with the velocity time integral (over one cardiac cycle) to compute stroke volume. Frequency analysis of the velocity waveform can be used to compute heart rate. Recent studies have used high-frequency (43–55 MHz) ultrasound, referred to as ultrasound biomicroscopy, due to the greater spatial resolution (30 μm). This increased spatial resolution enables measurement of chamber specific cardiac flows as well as velocity measurements in small vessels such as the aortic arches (Table 3; Phoon et al., 2000; Zhou et al., 2003; Butcher et al., 2007a; Hu et al., 2009; Oosterbaan et al., 2009).

**Table 3:** **Peak velocity measurements in normal embryos**.

**Animal**	**Stage**	**Location**	**Peak velocity (cm/s)**	**Technique**	**References**
Chick	HH25	Atrioventricular canal	17.1	Ultrasound	Butcher et al., 2007a
Chick	HH21	Inflow tract	5.0	Ultrasound	Oosterbaan et al., 2009
		Outflow tract	6.1		
Mouse	day 14.5	Mitral	34.4	Ultrasound	Zhou et al., 2003
		Tricuspid	33.4		
Chick	HH18	Outflow tract	7.8	Doppler-OCT	Rugonyi et al., 2008
Mouse	day 9.5	Dorsal aorta	0.8	Doppler-OCT	Larina et al., 2008
Quail	HH14	Inflow tract	2.62	Doppler-OCT	Jenkins et al., 2010
		Outflow tract	5.1		
Chick	HH17	Vitelline	0.32	Doppler-OCT	Davis et al., 2009
Chick	HH15	Ventricle	2.6	PIV	Vennemann et al., 2006
Chick	HH18	Vitelline	0.08	PIV	Poelma et al., 2008
Chick	HH18	Vitelline	0.1	OCT-PIV	Chen et al., 2012

OCT, another echo-based imaging modality, can produce higher resolution Doppler velocity measurements, though it has less depth of view. OCT uses low-coherence infrared light source and relies on interferometry to determine the location of back-reflected light. OCT measures a 1D velocity component, which must be combined with the angle between the beam and vessel centerline in order to obtain the true velocity. The maximum velocities measured with Doppler-OCT are limited by the scanning frequency of the OCT system, however phase unwrapping algorithms can be used to correct for slower scan rates (Davis et al., 2009). The resolution of OCT is such that it can measure velocity profiles, which is advantageous for calculating hemodynamic loading.

Apart from Doppler methods, micro particle image velocimetry (μPIV) has been used to measure blood flow velocities in chick and zebrafish embryos. μPIV uses cross-correlation between two consecutive images to calculate local displacements. In chick embryos, erythrocytes or injected fluorescent microspheres have been used as the seeding particles (Poelma et al., 2012). At high magnification, however, the use of red blood cells as tracer particles significantly underestimated the flow velocity (Poelma et al., 2012). Zebrafish experiments required transgenic embryos expressing green and red fluorescent proteins in endothelial and red blood cells, respectively (Chen et al., 2011). A high-speed camera integrated with a microscope or high speed scanning confocal are typically used to capture the source images. Unlike the previous Doppler methods, μPIV generates a field of 2D velocity vectors, which can identify flow structures such as vortices. OCT can be used as the imaging source for μPIV, as its speed and spatial resolution is sufficient to capture moving red blood cells. This technique, OCT- μPIV, has been applied to chick embryo vitelline vessels (Chen et al., 2012).

**Figure 1:**
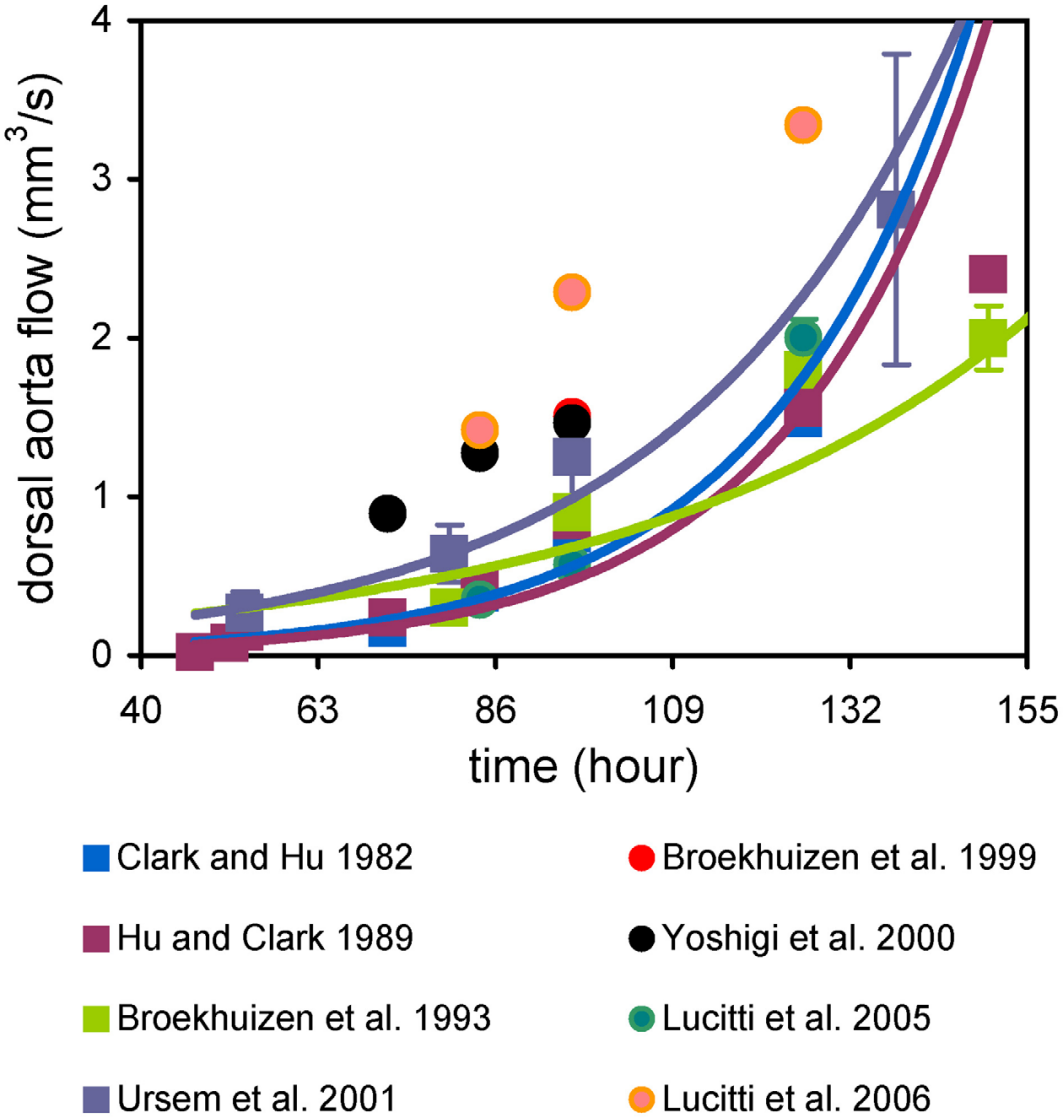
**Cardiac output increases exponentially during development**. Flow was measured using pulsed-Doppler ultrasound in chick embryos.

Cardiac output increases exponentially during embryonic development (Figure 1). Dorsal aortic flow, measured with 20 MHz directional pulsed Doppler ultrasound, is typically given as cardiac output, though it ignores cerebral perfusion (Clark and Hu, 1982; Hu and Clark, 1989; Broekhuizen et al., 1993, 1999; Yoshigi et al., 2000; Ursem et al., 2001; Lucitti et al., 2005, 2006). While this exponential rise in cardiac output is consistently observed, the reported values vary among researchers and depend upon reporting instantaneous peak or mean values (Figure 1). This variation may be due to measurement technique and experimental conditions. Using ultrasound biomicroscopy it is possible to investigate ventricular filling patterns. Similar to the mature heart, ventricular filling is biphasic in the embryo (Zhou et al., 2003; Butcher et al., 2007a; Oosterbaan et al., 2009). Peak velocity during passive ventricular filling increases exponentially during mouse embryonic development, while peak velocity during active filling changes logarithmically (Ishiwata et al., 2003; Zhou et al., 2003). Interestingly, in early stage chick embryos (HH17–23), the peak velocity during passive ventricular filling decreased (Butcher et al., 2007a; Oosterbaan et al., 2009). This trend suggests that maturation of myocardial relaxation occurs differently in chick and mouse embryos. Doppler ultrasound has been used to measure pulse wave velocity in the chick dorsal aorta via a dual-channel velocimeter. Pulse wave velocities ranged from 60 to 100 cm/s in embryos from HH21 to 27 (Lucitti et al., 2006).

**Figure 2:**
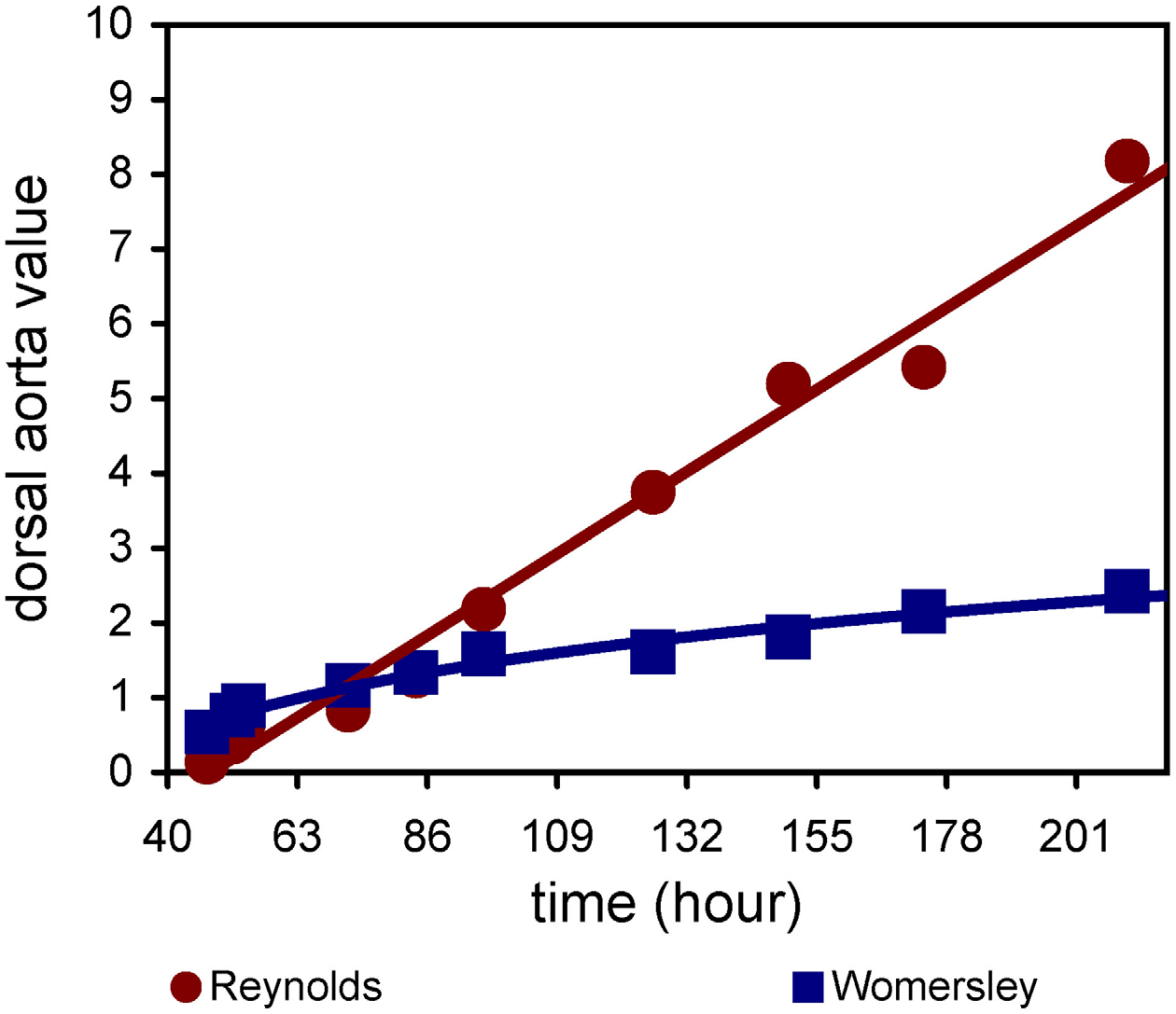
**Reynolds and Womersley numbers in the chick embryo dorsal aorta**. Flow and diameter data are based on Hu and Clark (1989), Broekhuizen et al. (1993). Viscosity is taken from Al-Roubaie et al. (2011). Blood density is assumed to be 1025 kg/m^3^.

Velocity measurements obtained using Doppler-OCT are similar to ultrasound (Table 3). Experiments with mouse and chick embryos demonstrate the utility of Doppler-OCT to obtain blood flow velocities at various locations, including the outflow tract and dorsal aorta (Larina et al., 2008; Rugonyi et al., 2008; Jenkins et al., 2010). μPIV analysis of flow within the HH15 chick ventricle revealed a laminar regime (peak Reynolds number 0.5), but an asymmetric velocity profile skewed toward the inner curvature of the heart (Vennemann et al., 2006). This skewed profile indicated a region of increased WSS at the inner curvature, consistent with WSS maps computed from μPIV and OCT data, as described below. Additional μPIV studies showed that blood flow within the embryonic cardiovascular system is laminar and quasi-steady, with low Reynolds (Re) and Womersley (Wo) numbers (Figure 2; Poelma et al., 2008). In the chick embryo dorsal aorta, Re and Wo range between 0.1 and 9 and 0.5 and 3, respectively, between HH12 and 36, compared to values of Re 1000 and Wo 40 in the mature human aorta. Computing Re and Wo numbers requires blood viscosity, which can differ significantly between embryonic and mature blood. In mature mammals, blood exhibits non-Newtonian behavior, in which the apparent viscosity decreases nonlinearly with increasing shear rates (Fung, 1997). This non-Newtonian rheology is primarily due to the presence of red blood cells, which are biconcave and deformable. Embryonic red blood cells, however, are spherical and make up a smaller percentage of the blood volume (Rychter et al., 1955). Rheological studies of HH24–34 chick embryonic blood revealed that hematocrit increases linearly, however the embryonic blood behaved as a Newtonian fluid throughout the investigated stages (Al-Roubaie et al., 2011). Hematocrit was also shown to be a good predictor of blood viscosity in the chick embryo.

An advantage of both OCT and μPIV techniques is the capability to obtain a velocity profile. These methods can therefore be used to compute the important biomechanical factor, WSS. WSS is defined as the shear rate (velocity gradient at the wall) multiplied by blood viscosity. For straight vessels, it is simple to compute the shear rate. However, for curved vessels, the vector normal to the wall must be obtained. μPIV methods performed in 2D can compute the normal vector from the microscopy image (Vennemann et al., 2006). OCT has the advantage of simultaneously acquiring structural and velocity data. Reconstructing the embryonic heart or vessel in 3D can give the wall normal vector, thus enabling WSS calculation. This approach has been used to obtain WSS levels in the early heart tube and vitelline veins (Davis et al., 2009; Jenkins et al., 2010; Peterson et al., 2012). Note that 4D reconstructions must be applied for the beating heart tube. A study using scanning μPIV to acquire a 3D flow field found WSS levels between 1 and 3 Pa in the outflow tract of an HH17 chick embryo (Poelma et al., 2010). An OCT-based study of the HH14 quail heart showed a region of high WSS (8 Pa) at the inner curvature of the outflow tract compared to other areas of the heart (2–3 Pa) (Peterson et al., 2012). These experimentally measured WSS values agree with computational modeling approaches (Table 4). OCT and μPIV techniques provide spatially resolved WSS maps, which can be coupled with local growth maps to investigate relationships between WSS and CV growth.

**Table 4:** **Peak WSS values in normal embryos**.

**Animal**	**Stage**	**Location**	**Peak WSS (Pa)**	**Technique**	**References**
Quail	HH14	Heart	8.40	OCT	Jenkins et al., 2010
Quail	HH14	Heart	8.10	OCT	Peterson et al., 2012
Chick	HH17	Outflow tract	3.00	PIV	Poelma et al., 2010
Chick	HH17	Atrioventricular canal	1.93	CFD	Yalcin et al., 2011
	HH23		7.83		
	HH27		25.01		
	HH30		28.72		
Chick	HH18	Aortic arches	5.47	CFD	Wang et al., 2009
	HH24		9.56		
Chick	HH16	Outflow tract	1.82	CFD	Bharadwaj et al., 2012
	HH23		5.82		
	HH27		23.61		
	HH30		53.61		
Chick	HH18	Outflow tract	6.00	CFD	Liu et al., 2012
Chick	HH17	Outflow tract	3.20	PIV	Poelma et al., 2010

### Pressure, resistance, and impedance

**Figure 3:**
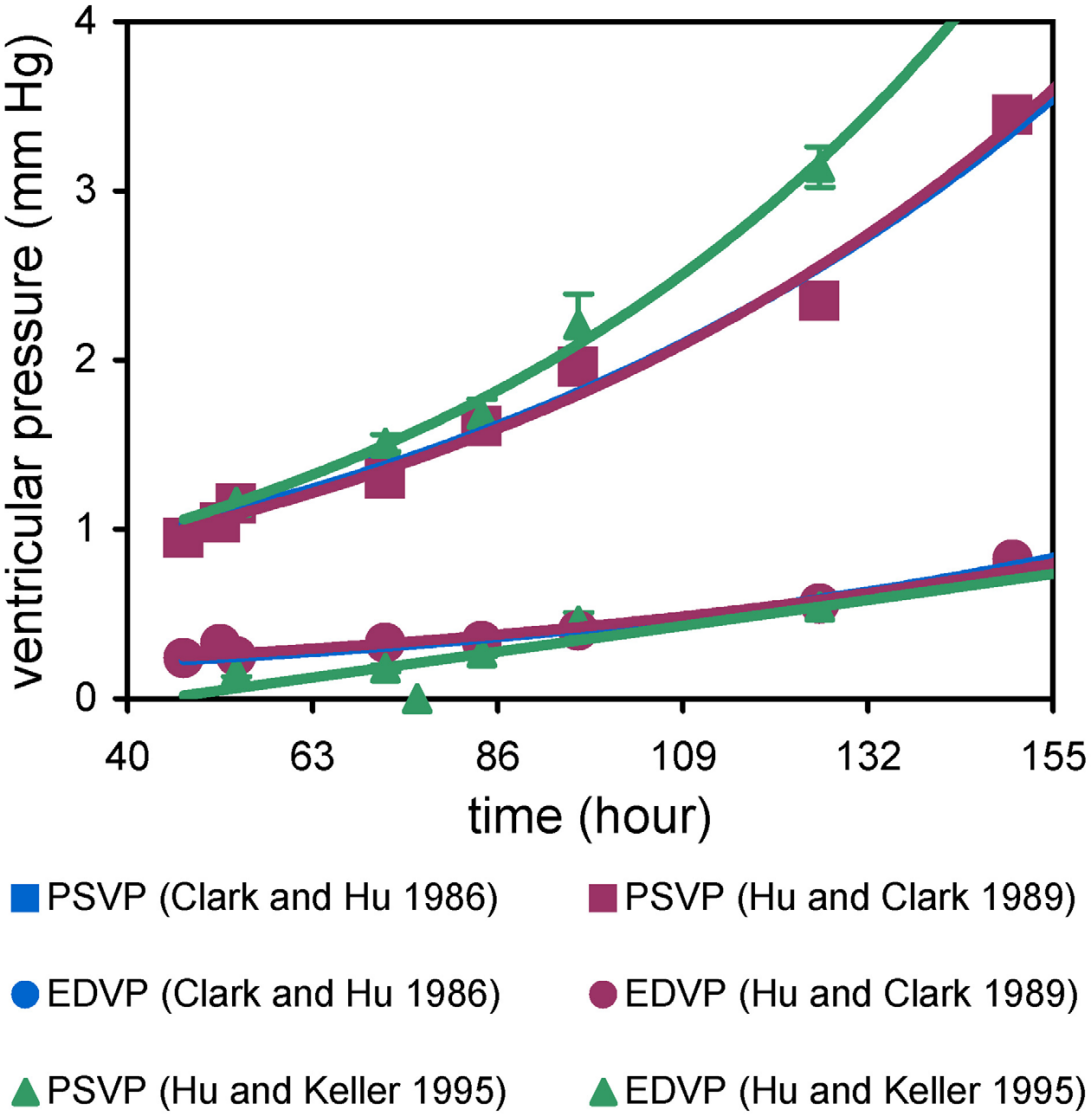
**End diastolic and peak systolic ventricular pressures (EDVP, PSVP)**. PSVP increases faster than EDVP, resulting in an increase in pulse pressure.

Intraventricular and intravascular pressure measurements in the embryo are performed with a servo-null transducer and drawn micropipette electrode (Falchuk and Berliner, 1971; Heineman and Grayson, 1985). End-diastolic and peak systolic ventricular pressures in chick and mouse embryos increase exponentially with developmental age, though peak systolic pressure increases more rapidly (Figure 3; Clark et al., 1986; Hu and Clark, 1989; Ishiwata et al., 2003). Thus, the pulse pressure between end-diastole and peak systole increases over development. Ventricular pressure waveforms in the embryo are similar to the mature heart, which suggests that endocardial cushions function as heart valves. Simultaneous atrial and ventricular pressure measurements in the chick embryo examined the atrioventricular (AV) pressure gradient, which remained approximately 0.2 mmHg from HH16 to 21, but increased sharply to 0.6 mm by HH27 (Hu and Keller, 1995). The sharp increase may be related to septation of the atrium and AV canal. This study also found that atrial pressure exceeds ventricular pressure throughout ventricular filling (Hu and Keller, 1995) suggesting that the developing AV cushions have some passive resistance during diastole. A pressure gradient also exists between the embryonic ventricle and descending aorta, consistent with a combined resistance of the outlet cushions and the aortic arches. Pressure measurements of vitelline arteries in chick embryos revealed a linear increase (Clark and Hu, 1982; Hu and Clark, 1989). The linear increase in arterial pressure coupled with exponential rise in cardiac output produces a geometric decrease in vascular resistance (Table 5; Hu and Clark, 1989; Lucitti et al., 2005). These trends occur concurrently with increasing ventricular size and weight, myofiber organization, and increased ventricular trabeculation (Sedmera et al., 1999, 2000; Tobita et al., 2005; Baker et al., 2008). In section Hemodynamic Intervention Models of this review, we discuss how experiments that perturb embryonic blood flow demonstrate that these hemodynamic and structural changes are linked.

**Table 5:** **Vascular hemodynamic properties of the normal chick embryo**.

**Stage**	**Vascular resistance (mmHg/mm^3^/s)**	**Cardiac work (nJ)**	**Systolic pressure (mmHg)**	**Total hydraulic power (nW)**
HH18	2.95	9.3	1.14	48
HH21	2.05	20.0	1.48	108
HH24	1.21	41.3	1.82	296
HH27	0.83	89.3	2.51	1054
HH29	0.63	138.9	3.04	2203

*References: Hu and Clark, 1989; Zahka et al., 1989*.

Pressure measurements have been combined with video microscopy to analyze pressure-volume relationships in the chick embryo. Initially, pressure-area loops were used, where epicardial ventricular cross-section area was measured from video recordings. These pressure-area loops resembled mature pressure-volume loops, with clearly observed diastolic filling, isometric contraction, ejection, and isometric relaxation phases (Keller et al., 1991). Isometric contraction times increased linearly from HH16 to HH24 while isometric relaxation times remained similar. This technique was expanded to generate pressure-volume loops by using an ellipsoid geometry model to estimate ventricular volume based on area (Stekelenburg-De Vos et al., 2005). These pressure-volume loops have been used to compute contractility and compliance properties of the embryonic ventricle. Additionally, the presence of isometric contraction and relaxation provide further evidence that the endocardial cushions function as valves.

Simultaneous velocity and pressure measurements at the dorsal aorta allow for analysis of impedance and hydraulic power. In the chick embryo, the first, second, and third impedance moduli all decreased from HH18 to 29 (Zahka et al., 1989). This decrease is likely due to growth and remodeling of the dorsal aorta and vascular bed. Total hydraulic power of the chick embryo CV increased 50-fold from 48 to 2600 nW between HH18 and 29 (Table 5), while the proportion of hydraulic power due to pulsatile flow increased from 28 to 65% (Zahka et al., 1989). Remarkably, the embryonic vascular bed can be accurately represented by a 3 element Windkessel model, similar to the mature circulation (Yoshigi et al., 2000) and the embryonic vasculature rapidly adjusts impedance to loading conditions, similar to the ability of the ventricle to rapidly adjust to changes in preload and afterload.

Structural remodeling of large arteries and veins, from thin mesenchymal sheaths to organized, multi-layer lamellae of circumferentially oriented smooth muscle cells and extracellular matrix components such as elastin and collagen occurs along with the exponential rises in blood pressure and flow (Deruiter et al., 1993; Hungerford et al., 1996; Waldo et al., 1996; Majesky et al., 2011). Shear stress and cyclic transmural stress and strain are important biomechanical factors regulating microstructural remodeling; in mouse embryos with impaired cardiac contractility, blood vessels appeared disorganized with separated endodermal and mesodermal layers (Huang et al., 2003; May et al., 2004). Large arteries near the heart have high elastin content, which allows them to elastically deform under high pressure, creating a windkessel effect necessary to maintain blood flow at the physiologic pressure range. In mouse embryos lacking elastin, deformation of the aortic wall, measured with M-mode ultrasound, was 3 times less vs. controls at day 18 (Wagenseil et al., 2009). The production of extracellular matrix components is indicative of smooth muscle maturation, and the time-course of extracellular matrix gene expression follows hemodynamic trends during embryonic and postnatal development: expression is high during rapid increases in pressure and flow and minimal once a stable hemodynamic state is achieved (Wagenseil and Mecham, 2009). Additional discussion of the development and remodeling large arteries can be found in previously published reviews (Ribatti, 2006; Cheng and Wagenseil, 2012; Seidelmann et al., 2014).

### Placental circulation and maternal stress

The exchange of nutrients, gas, water, and metabolic waste during embryonic and fetal life is carried out through the extraembryonic umbilical circulation. In placental mammals, the umbilical circulation connects with the placenta, which is also connected to the mother via the uterine circulation, and exchange between fetal and maternal blood occurs across various plasma membranes (Desforges and Sibley, 2010). Other amniotes such as birds and reptiles rely on the vitelline circulation to receive nutrients from the yolk and the allantois for gas and nitrogenous waste exchange. These external circulation pathways are eliminated after birth, but have important roles in shaping the hemodynamic environment of the embryo. Umbilical blood flow increases exponentially over gestation, following both fetal growth rates and increases in cardiac output (Rudolph and Heymann, 1970; Reynolds and Redmer, 1995). Throughout the last half of gestation in humans, umbilical blood velocity increases and resistance decreases (Gadelha Da Costa et al., 2005). This increased blood flow allows for greater placental transport capacity to support the geometric increase in fetal oxygen demand, for example a 16-fold increase in oxygen uptake in the bovine fetus from mid-to-late gestation (Reynolds et al., 1986).

Sheep models of compromised pregnancy, including nutrient deprivation, nutrient excess, environmental heat stress, and hypoxic stress all lead to low placental weight and decreased uterine and umbilical blood flow (Krebs et al., 1997; Regnault et al., 2003; Wallace et al., 2005), similar to human conditions. Fetal oxygen and nutrient uptakes are reduced in these models. In humans, restricted uterine and umbilical blood flows resulted in fetal hypoxia, hypoglycemia, and asymmetric organ growth (Pardi et al., 1993; Ferrazzi et al., 2000). Placental insufficiency is associated with the majority of intrauterine growth restriction, with etiologies including fetal genetic diseases or congenital malformations, maternal hypertensive disorders causing reduced uteroplacental flow, decreased oxygen carrying capacity of the mother, and malnourishment (Hendrix and Berghella, 2008). These conditions lead to decreased systolic and diastolic cardiac function in the fetus as well as free wall hypertrophy and increased coronary blood flow (Bahtiyar and Copel, 2008). Thus, maternal stresses causing impaired uterine blood flow and compromised placental function and circulation directly affect fetal growth (Reynolds et al., 2005, 2010). Removal of the umbilical circulation after birth by cord clamping is additionally indicated as a potential source of hemodynamic disruption, as it can limit transfer of placental blood and prostaglandins to the neonate (Sommers et al., 2012; Bhatt et al., 2013; Hutchon, 2013; Raju, 2013; Yigit et al., in review).

## Computational modeling approaches

Development of computational models is vital to investigating the biomechanics of CV development. Models are used to calculate data that cannot be measured experimentally and enable incremental studies where a single parameter can be methodically altered. Aspects of the embryonic CV have been modeled using a variety of methods, including computational fluid dynamics (CFD), continuum solid mechanics, and lumped parameter models. Experimental data is required to train and test the results of computational models and is often needed for initial inputs. Insights gained from modeling can, in turn, inspire new experiments. Thus, experiments and models complement each other and are both needed to study embryonic CV development.

**Figure 4:**
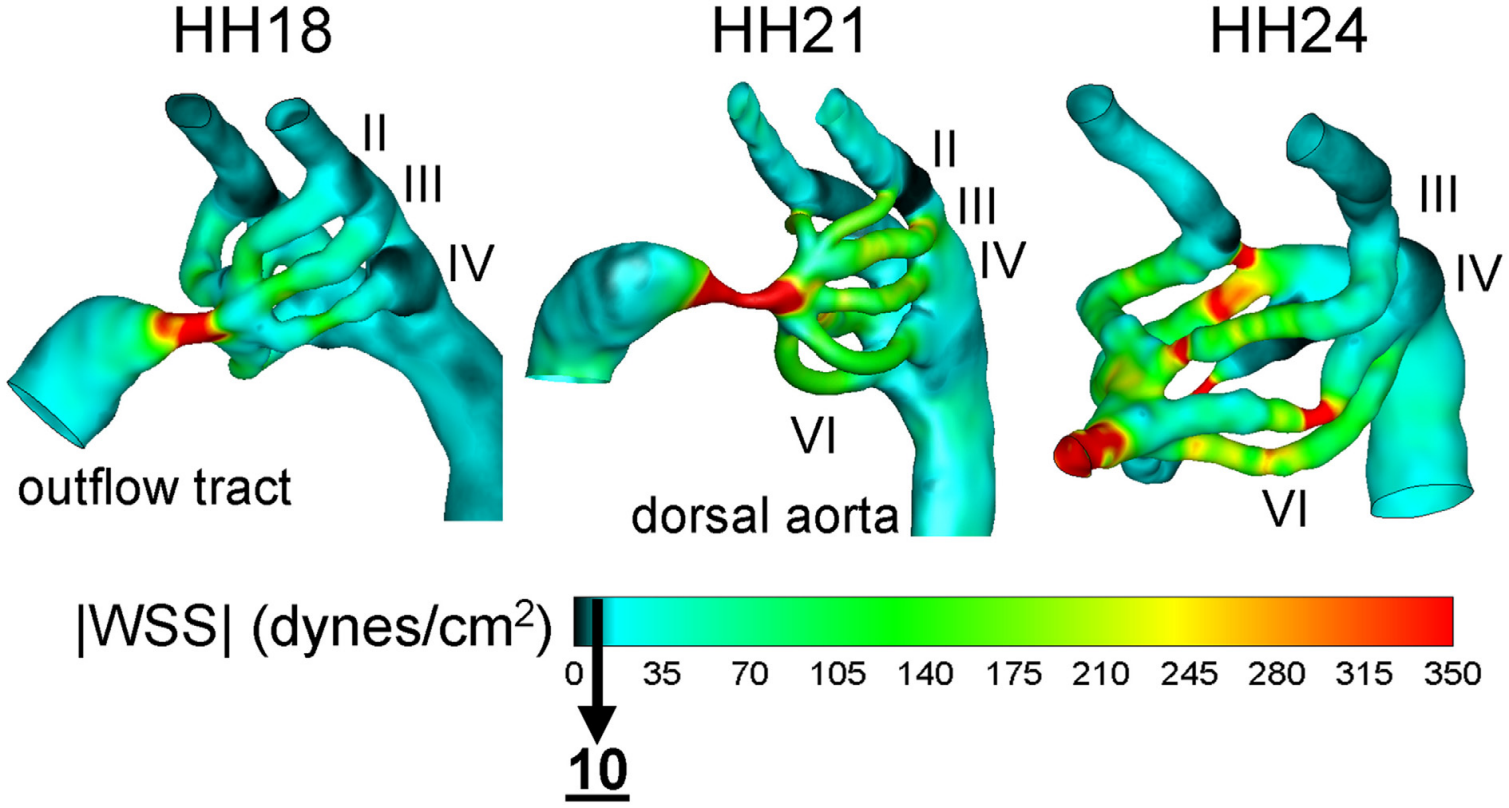
**Aortic arch peak flow wall shear stress (WSS) distribution for HH 18, 21, and 24 chick embryos**. WSS is computed based on a CFD model. Aortic arches are viewed from a left oblique angle and numbered by roman numeral (II-VI). In order to display a broader range, a peak WSS of 350 dynes/cm2 is shown. Adapted from Wang et al. (2009), Kowalski et al. (2013).

CFD is widely used to analyze hemodynamics at a higher level than Doppler velocimetry or μPIV can provide. Doppler techniques are not suited for complex flow fields and μPIV requires vessels that are accessible and not obscured. CFD models overcome these limitations by providing 3D data. Model geometries are often obtained from imaging techniques described in Section Introduction. Pressure and velocity measurements are used to set realistic boundary conditions. Depending on the model objectives, blood flow can be at he mean steady-state or pulsatile. CFD models of flow through the AV canal of chick embryos at different stages of development demonstrated that WSS increases monotonically and is higher on the left side of the heart (Table 4; Yalcin et al., 2011; Bharadwaj et al., 2012). A CFD model of the HH18 chick embryo outflow tract that incorporates wall motion was developed based on 4D OCT measurements and revealed that WSS is highest at the outer curvature and on the endocardial cushions (Liu et al., 2011). Models of blood flow through the chick embryo aortic arches quantified flow patterns and cardiac output distribution at HH18, 21, and 24 (Figure 4; Wang et al., 2009; Kowalski et al., 2013). These models were coupled with experimental measurements of aortic arch diameter and revealed a correlation between asymmetric flow distributions and asymmetric growth. Further, WSS was shown to become elevated at the intermediate, transitional stage HH21 and then return to previous levels by HH24. Each of these models uncovered hemodynamic features that could not be captured by experiment alone. By combining experimental and modeling techniques, new insights are gained into the biomechanical influence on CV growth and morphogenesis.

Computational models that recapitulate or predict embryonic CV growth are emerging and have been developed with some success. A common approach is to use a stress-based growth law that assumes a hypothetical homeostatic stress state. A mechanistic growth field is generated in order to reach this equilibrium state. This approach was used to develop a model of growth and remodeling of the heart from its first beat to maturity (Taber and Chabert, 2002). The model incorporated myocardial trabeculation and compaction by applying temporal changes to material coefficients. When applying a radial cut, the opening angle predicted in the model agreed reasonably with experimental data. In a model of AV valve growth, fluid dynamics were incorporated into a stress-based model, where flow simulations occurred after growth integration in order to compute the new biomechanical load (Buskohl et al., 2012). This model showed that pressure distribution drove leaflet elongation while shear regulated structural remodeling. Aortic arch selection was modeled using a CFD-coupled optimization framework, where individual aortic arch diameters were varied to minimize total power and maximize diffusive capacity (Kowalski et al., 2012). The model predicted HH18 aortic arch diameters to within one standard deviation of experimental values. Further research will lead to predictive models that can be used to test the progression of CHD.

## Hemodynamic intervention models

Early experiments performed by Rychter sought to disrupt blood flow by occluding various segments of the CV system through ligation or clipping (Rychter, 1962; Rychter and Lemez, 1965). Though primarily qualitative descriptions of the resulting morphological changes, these experiments provided direct evidence that hemodynamic disruptions generate CV defects. Currently, mechanical interventions, genetic mutations, and pharmacological manipulations are used in conjunction with the quantitative techniques described above to provide further insight into the biomechanical regulation of CV morphogenesis. The size and access of the chick embryo makes it an ideal model for surgical interventions, while genetic models are more commonly founded on mouse and zebrafish. The sections below describe a variety of interventions designed to perturb embryonic hemodynamics. These examples are not exhaustive, but provide background on some of the more commonly used approaches.

### Vitelline vein ligation (VVL)

Occlusion of a major vitelline vein by ligation or placement of a clip mimics reduced placental blood flow and is one of the most widely used interventions to modify blood flow. Performed on HH17 chick embryos, cardiac defects observed at HH36–45 included ventricular septal defects, AV canal defects, bicuspid aortic and quadricuspid pulmonary valves, interrupted aortic arch, double aortic arch, and hypoplastic pulmonary arteries (Hogers et al., 1999). VVL immediately disrupts intracardiac flow patterns, particularly at the AV canal and outflow tract (Hogers et al., 1999). Cardiac output, peak flow in the dorsal aorta, and heart rate all reduced immediately following right lateral VVL; heart rate and cardiac output returned to normal levels within 3 h (Stekelenburg-De Vos et al., 2003). After 1.5 days of chronic ligation, only peak acceleration in the dorsal aorta was reduced; dorsal aorta diameter, peak and mean velocity and flow, stroke volume and dorsal aorta pressure were normal (Broekhuizen et al., 1999). By 5.5 days after ligation, stroke volume and peak and mean dorsal aorta velocity and flow were increased, which also coincided with a ventricular septal defect in 6 out of 13 embryos (Broekhuizen et al., 1999). After placement of a clip on the right vitelline vein at HH17, analysis of pressure-volume loops at HH21 showed reduced contractility and compliance, which may indicate impaired myocardial development (Stekelenburg-De Vos et al., 2005).

These hemodynamic changes have been linked to altered expression of Kruppel-like factor 2 (KLF2), endothelin-1 (ET-1) and endothelial nitric oxide synthase (NOS-3) within the embryonic heart 1 h after right lateral VVL (Groenendijk et al., 2005). Increased KLF2 and NOS-3 expression was present at the narrowing of the AV canal, the junction between the AV canal and outflow tract cushions, and the upstream slope of the outflow tract, while KLF2 was decreased in downstream AV canal and the descending aorta. ET-1 was downregulated at the downstream slope of the AV canal and in the junction between the AV canal and outflow tract cushions and was increased in the dorsal aorta. Analysis of blood flow in the outflow tract using μPIV showed that the WSS distribution at peak systole increased after VVL (Egorova et al., 2011). The expression patterns in the outflow tract (KLF2 and NOS-3 upregulated) are consistent with increased WSS. Additionally, TGFβ /ALK5 signaling was increased in endothelial cells lining the outflow tract cushions (Egorova et al., 2011). Further studies on *ex vivo* cultures of embryonic mouse endothelial cells demonstrated shear-mediated ALK5 signaling (Egorova et al., 2011). These VVL models provide some of the strongest evidence for biomechanical regulation of CV development, especially the role of WSS in modifying gene expression and signaling.

### Outflow tract banding (OTB)

Increasing afterload by tightening a suture around the embryonic outflow tract has generated valuable insight into the role of ventricular pressure on myocardial remodeling (Clark et al., 1989). OTB accelerates trabecular remodeling and myocardial compaction, resulting in a thickened compact layer (Sedmera et al., 1999). Additionally, myofiber organization is accelerated in OTB chick embryos (Tobita et al., 2005). Ventricular septal defects with either double outlet right ventricle or persistent truncus arteriosus occur after OTB as well as valvular anomalies (Sedmera et al., 1999). Mechanical testing of left ventricle tissue sections showed significantly stiffer stress-stain relations 2 days after OTB (Miller et al., 2003). Ventricular stiffening was also observed in pressure-inflation tests of excised hearts after OTB, which further showed that the right ventricle normalized end-diastolic strain rather than stress while the left ventricle normalized both end-diastolic strain and stress (Tobita et al., 2002). Structural and Doppler OCT measurements of HH18 OTB chick embryos demonstrated restricted outflow tract motion and increased blood velocity (110 mm/s OTB vs. 78 mm/s normal peak velocity) (Rugonyi et al., 2008).

### Left atrial ligation (LAL)

**Figure 5:**
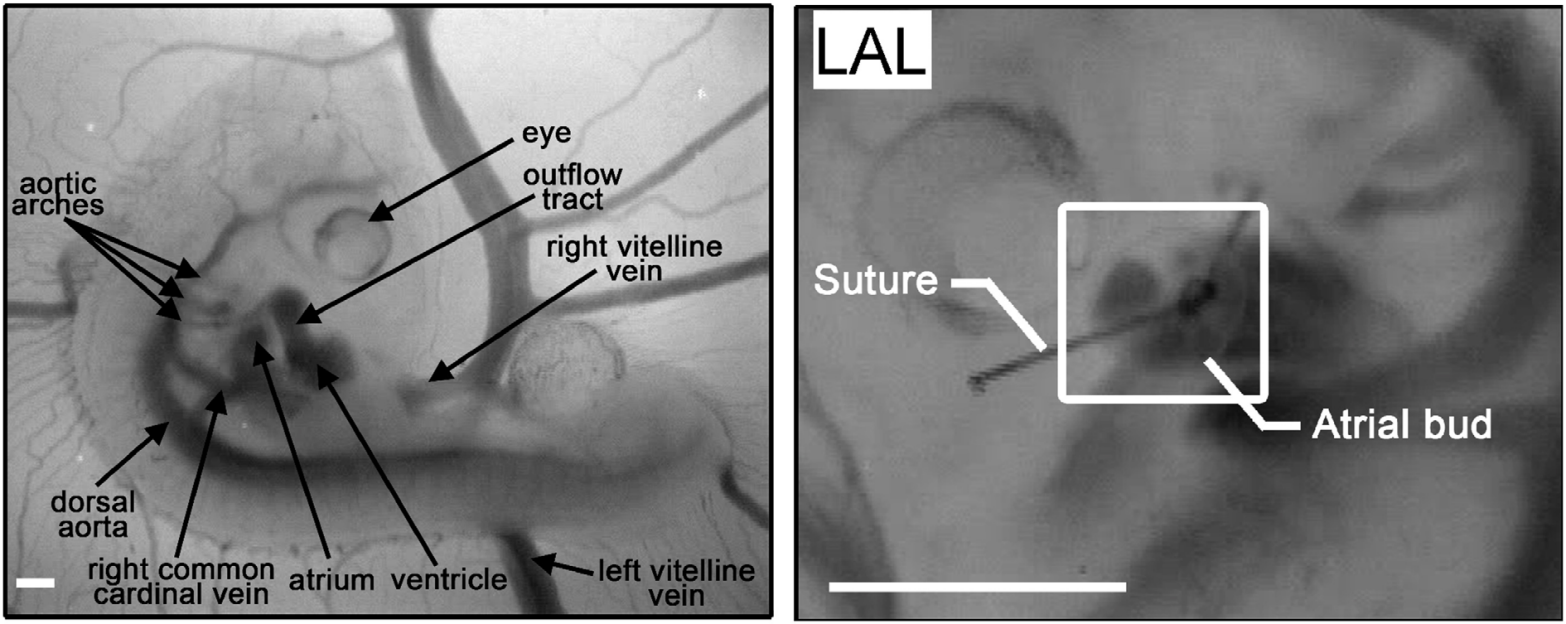
**HH21 chick embryo model**. **Right panel:** a normal embryo viewed from the right lateral with major vascular structures labeled. **Left panel:** a repositioned left side up embryo after LAL viewed from the left lateral to illustrate the ligated left atrium. Scale bars = 500 μm.

Hypoplastic left heart syndrome (HLHS), is a rare but serious congenital heart defect, marked by an underdeveloped and nonfunctioning left ventricle and hypoplastic ascending and transverse aorta in association with stenosis or atresia of the mitral and/or aortic valves, and intra-uterine compensatory enlargement of right sided cardiac structures (Friedman et al., 1951; Noonan and Nadas, 1958). Left atrial ligation (LAL) in the chick embryo remains the only long term prenatal animal model of HLHS (Rychter, 1962). The presumptive left atrium is tied off, disrupting inflow to the left side of the embryonic ventricle (Figure 5). Immediately following LAL, intracardiac flow patterns shift ventrally (Kowalski et al., 2014). Downstream cardiac pressures are unchanged, but cardiac output is transiently reduced, returning to normal levels by 32 h post-LAL (Lucitti et al., 2005). Abnormal remodeling of the ventricular myocardium is observed after 2 days, including decreased myocardial volume, accelerated trabecular compaction, and delayed changes in transmural myofiber angle (Sedmera et al., 1999; Tobita et al., 2005). Circumferential and longitudinal strain increases in both ventricles after LAL, while the onset of preferential circumferential strain patterns in the right ventricle are accelerated and the preferential longitudinal strain patterns in the left ventricle are abolished (Tobita and Keller, 2000). At the cellular level, LAL results in reduced proliferation in the left ventricular compact layer and trabeculae, decreased FGF-2 and PDGF-B expression throughout the heart, a greater number of apoptotic cells in the right AV cushions, and increased microtubule density in the left ventricular compact layer (Schroder et al., 2002; Sedmera et al., 2002). Downstream vessels are also affected in LAL; flow distribution within the aortic arches is disrupted, and defects including aortic arch hypoplasia and interrupted aortic arch are observed as early as 32 h after ligation (Hu et al., 2009).

### Other hemodynamic interventions

Altering heart rate is a simple method for disrupting CV function and examines how cycle length affects cardiac performance. In one study, heart rate altered by applying a heated or cooled metal probe to the sinus venosus of HH21 chick embryos, with simultaneous pressure-volume loop analysis (Casillas et al., 1994). End diastolic volume and stroke volume varied linearly with cycle length whereas end systolic pressure varied inversely. Interestingly, end diastolic pressure was not dependent on cycle length, and the heart was able to acutely increase stroke volume without an increase in developed ventricular pressure. As no functioning autonomic nervous system exists at HH21, the vascular response may be induced by flow, stretch, or endocrine mechanisms.

Vitelline artery ligation (VAL) is a method for increasing peripheral vascular resistance in chick embryos. VAL performed at HH21 resulted in reduced stroke volume and cardiac output, increased total vascular resistance, larger first harmonic and characteristic impedances, decreased arterial compliance, and transient changes in pulse pressure, hydraulic power, and oscillatory power, which all normalized by HH27 (Lucitti et al., 2005). Structural effects of VAL were investigated through pressure-inflation tests of excised dorsal aortae, which revealed an increased stiffness in VAL embryos (Lucitti et al., 2006). Smooth muscle α-actin and collagen-III were present in greater amounts in the dorsal aortae of VAL embryos, demonstrating a remodeling response to the altered load (Lucitti et al., 2006).

Blood flow can be entirely eliminated in chick embryos by removing the heart at HH12. These embryos showed underdeveloped cranial and cervical regions and mesenchymal edema at HH17 as well as abnormal yolk sac vasculature (Manner et al., 1995). Incubation in hyperoxic conditions achieved normal cervical growth and mesenchyme, but did not recover yolk sac remodeling, suggesting an early metabolic role for blood flow in cranial development whereas vascular remodeling depends primarily on the mechanical factors induced by blood flow (Manner et al., 1995). In a separate study, obliteration of blood flow in chick embryos was achieved by incubating with an open shell window, dehydrating the embryo, and resulted in arrested remodeling of the yolk sac vasculature with no arterial-venous differentiation (Le Noble et al., 2004). This study further demonstrated through a vitelline artery ligation model that arterial markers neuropilin 1 and ephrinB2 were downregulated on the ligated side while venous markers neuropilin 2 and Tie2 were upregulated (Le Noble et al., 2004). Upon removing the ligation, expression of neuropilin 1 returned. Thus, arterial-venous differentiation was not only influenced by flow conditions, but also adaptable to modified flow over a certain timeframe (ligations lasting more than 6 h did not return neuropilin 1 expression) (Jones et al., 2006). Flow occlusion in zebrafish embryos was achieved by inserting microbeads at the inflow or outflow regions, which resulted in abnormal cardiac chamber morphology and disrupted valve formation, demonstrating the importance of shear stress in the development of these structures (Hove et al., 2003).

Lowering blood viscosity by reducing hematocrit is an interesting approach to decrease WSS throughout the CV. In mouse embryos, primitive erythroblasts were sequestered in blood islands using polyacrylamide to reduce viscosity and therefore WSS (Lucitti et al., 2007). Under this decreased WSS, the yolk sac failed to remodel. Injecting viscous hetastarch was able to recover normal remodeling, however. These results suggest that WSS is the primary factor, rather than oxygen or nutrient delivery, for vascular remodeling in the embryo. In zebrafish embryos, blood viscosity was lowered by using morpholinos against *gata1* (no blood cells) and *gata2* (72% less blood cells) (Vermot et al., 2009). This modified viscosity changed the reverse flow fraction at the AV canal from 35% normally to 45% in *gata1* morphants and 17% in *gata2* morphants. At later stages, most *gata1* embryos had normal valves, while the majority of *gata2* morphants developed valve defects, suggesting that reverse flows are critical for valve remodeling. Genetic zebrafish models such as *silent heart (sih*−/−), which lacks a heartbeat, and *cardiofunk (cfk*−/−), which exhibits cardiac dilation offer novel methods to investigate embryonic development under low- to no-flow conditions (Bartman et al., 2004).

Pharmacologic models have been used to generate transient alterations in embryonic hemodynamics by altering ventricular function and/or arterial afterload. Calcium channel blockade (verapamil) reduces ventricular contractility in the immature myocardium (Clark et al., 1991; Tenthorey et al., 1998), caffeine reduces ventricular function due to increased vascular impedance due to vasoconstriction (Bruyere et al., 1987, 1988; Momoi et al., 2008) and chronic hypoxia alters ventricular function due to mixed effects (reduced contractility and increased vascular resistance).

Computational modeling approaches discussed in Section Computational modeling approaches have been applied to investigate effects of the surgical interventions described above. Mechanical stress and strain was modeled in ventricle geometries obtained from normal, OTB, and verapamil suffused (pressure-underloaded) HH29 chick embryos (Buffinton et al., 2013). Flow in normal and *in silico* generated LAL HH21 heart geometries was modeled using CFD to examine flow patterns and WSS, though the static model was not sufficient to recapitulate experimental results (Kowalski et al., 2014).

## Summary and outlook

Hemodynamic forces such as WSS and pressure provide biomechanical cues that stimulate growth and remodeling of the CV system. Experimental techniques to quantify this environment include velocimetry, pressure measurement, and dynamic *in vivo* imaging. These methods have identified several trends in hemodynamic parameters over development. Computational models complement these experiments and provide further insights into the biomechanical regulation of CV morphogenesis. Interventions to test the CV response to perturbed flow on a healthy/normal functioning genetic pathway demonstrate the role of hemodynamics and uncover important aspects of how blood flow influences CV growth. As these techniques and models advance, so will the understanding of CV development.

There is still a need for greater quantification of CV morphology in both normal and under experimentally perturbed conditions. Live imaging techniques that allow time-lapse, long-term studies are a promising prospect to quantify growth on a local and temporal scale. Real-time growth data can, in turn, be used to train predictive computational models. The inclusion of cellular and molecular methods to study the biologic mechanisms of biomechanical regulation is also greatly needed. Studies performed under *in vivo* conditions, rather than isolated *in vitro* tests, will be important to determine how hemodynamic force is converted into tissue remodeling. Additionally, a greater investigation of the mechanical properties of CV tissues is required to fully understand the effects of structural remodeling. Developing these areas is vital to ensure future discovery.

Investigating the critical biomechanical loading thresholds that regulate local vascular morphogenesis and remodeling is another rapidly expanding landscape. We anticipate that the cardiac chambers and vascular regions have unique vulnerabilities to altered biomechanical stresses, directly contributing to the range of abnormal morphologies found in children with CHD. While we cannot predict, a priori, the amplitude or duration required to trigger these critical events, it is likely that the mechanical and temporal thresholds will correspond to the thresholds required to transduce changes in biomechanical loading in to altered cellular fates (proliferation, maturation, death).

Defining the role of biomechanics in CV development creates opportunities for new treatment strategies for CHD. The current understanding of flow and growth relationships has been translated as a rationale for cardiac intervention in the mid-gestation human fetus and intervention in humans has shown that restoration toward normal biomechanical loading partially restores the growth and remodeling of left heart structures (mitral valve, aortic valve, aorta), though recovery of fetal myocardial growth after fetal intervention remains suboptimal (Pekkan et al., 2008a,b; McElhinney et al., 2010). The application of science and engineering approaches to study embryonic biomechanics will further our understanding of these relationships, potentially improving the outcomes for fetal intervention of CHD.

### Conflict of interest statement

The authors declare that the research was conducted in the absence of any commercial or financial relationships that could be construed as a potential conflict of interest.
